# THE RELATED FACTORS ON HEALTH DEFICIENCIES IN NATIONAL NURSING HOMES USING PANEL LARGE DATASET

**DOI:** 10.1093/geroni/igad104.3653

**Published:** 2023-12-21

**Authors:** Juh Hyun Shin, Jinhyun Ahn, Soo-Kyoung Lee, Charlene Harrington, Sunwoo Jung, Yeonwoo Lee, Sanna Ali

**Affiliations:** George Washington University, Ashburn, Virginia, United States; Jeju National University, Jeju, Jeju, Republic of Korea; Seoul National University, Seoul, Republic of Korea; University of California, San Francisco, San Francisco, California, United States; Jeju National University, Jeju, Jeju, Republic of Korea; Jeju National University, Jeju, Jeju, Republic of Korea; The George Washington University, Washington, District of Columbia, United States

## Abstract

Health deficiencies are considered scientific research outcomes and should be reported as mandatory for all Medicare-Medicaid nursing homes (NHs). However, health deficiencies have been a concern for several decades. Previous studies have focused on regulatory deficiencies or used nurse staffing variables as control variables. This study investigated health deficiencies in relation to nurse staffing, facility size, and ownership across all Center for Medicare and Medicaid NHs in the United States. This study used secondary data analysis using Payroll-based Journal Daily Nurse Staffing data, Area Resource File, and LTCFocus. Data were merged with federal Medicare/Medicaid provider IDs as the basic unit of analysis for the 2019 fiscal year. A total of 7,802 NH data were analyzed using Poisson regression. The average total weighted health deficiencies survey score was 66.12. The average bed size was 114.02, where 74% were profit NHs. The average hours per resident day (HPRD) for each nurse staffing was as follows: 0.57 registered nurses, 0.86 licensed practical nurses, and 2.16 certified nurse aides. As total HPRD increases by 1 unit (exponential 0.83), it is likely that health deficiencies decrease by 17%. The bed size less than 60 (exponential 0.87) and not-for-profit NHs (exponential 0.09) was related with decreased health deficiencies of 13% and 91%. The worsened residents’ acuity was related with 2% more health deficiencies (exponential = 0.98). This large dataset analysis shows that professional nurse staffing is associated with superior NH quality of care with fewer deficiencies and supports the need for age-friendly long-term healthcare systems.

